# Diagnostic Role of Immunofluorescence Analysis in Primary Ciliary Dyskinesia-Suspected Individuals

**DOI:** 10.3390/jcm14061941

**Published:** 2025-03-13

**Authors:** Elif Karakoç, Rim Hjeij, Zeynep Bengisu Kaya, Nagehan Emiralioğlu, Dilber Ademhan Tural, Pergin Atilla, Uğur Özçelik, Heymut Omran

**Affiliations:** 1 Department of Histology and Embryology, Faculty of Medicine, Hacettepe University, 06230 Ankara, Turkey; kaya.zeynep@mayo.edu (Z.B.K.); patilla@hacettepe.edu.tr (P.A.); 2 Department of General Pediatrics, University Hospital Muenster, 48149 Muenster, Germany; rim.hjeij@ukmuenster.de; 3 Department of Neuroscience, Mayo Clinic, Jacksonville, FL 32224, USA; 4 Department of Pediatric Pulmonology, Faculty of Medicine, Hacettepe University, 06230 Ankara, Turkey; drnagehan@yahoo.com (N.E.); dilberademhan@gmail.com (D.A.T.);

**Keywords:** immunofluorescence, PCD, ODA, IDA, HSVM

## Abstract

**Background/Objectives**: Primary ciliary dyskinesia (PCD) (OMIM: 244400) is a hereditary, rare disorder with a high prevalence in Turkey due to a high rate of consanguinity. The disorder is caused by malfunctioning motile cilia and is characterized by a variety of clinical symptoms including sinusitis, otitis media and chronic obstructive pulmonary disease. This study presents the first assessment of the efficacy of immunofluorescence (IF) labeling for diagnosing PCD in Turkey by correlating IF with clinical observations when genetic data are scarce. **Methods**: We have a cohort of 54 PCD-suspected individuals with an age range of 5–27 years classified into two groups: group A with available genomic data (8 individuals) and group B with no available genomic data (46 individuals). We performed immunofluorescence analysis to confirm the pathogenicity of the variants in individuals with a prior genetic diagnosis and to confirm a PCD diagnosis in individuals with typical PCD symptoms and no genetic diagnosis. **Results**: All individuals had airway infections and displayed clinical symptoms of PCD. Our data revealed an absence of outer dynein arm dynein heavy chain DNAH5 in individuals with pathogenic variants in *DNAH5* and *DNAAF1* and in 17 other PCD-suspected individuals, an absence of nexin–dynein regulatory complex component GAS8 in 8 PCD-suspected individuals, an absence of outer dynein arm dynein heavy chain DNAH11 in 6 PCD-suspected individuals and an absence of radial spoke head component RSPH9 in 2 PCD-suspected individuals. Furthermore, the pathogenicity of *ARMC4* variants was confirmed by the absence of the outer dynein arm docking complex component ARMC4 and the proximal localization of DNAH5. **Conclusions**: Immunofluorescence analysis, owing to its lower cost and quicker turnaround time, proves to be a powerful tool for diagnosing PCD even in the absence of genetic data or electron microscopy results.

## 1. Introduction

Primary ciliary dyskinesia (PCD) (OMIM: 244,400) is an inherited rare disorder with a prevalence of approximately 1:10,000 to 1:20,000 and a mortality rate of around 5% among adults [1]. PCD is caused by dysfunction of motile cilia and is characterized by various clinical symptoms such as chronic airway illness, otitis media, sinusitis, hydrocephalus, hearing deficits and male infertility [2,3,4]. Additionally, 50% of individuals with PCD exhibit laterality defects such as situs inversus, and 6% are affected with congenital heart disease [3,5].

Cilia are hair-like organelles composed of microtubules capable of movement, driven by their distinct motor protein complexes [6]. The classical 9+2 axonemal structure of cilia consists of two microtubules located centrally (central pair apparatus) and nine doublets peripherally [7]. Outer dynein arms (ODAs) and inner dynein arms (IDAs) regulate ciliary motility [6]. The nexin–dynein regulatory complex (N-DRC) connects the peripheral microtubules, and, together with ruler proteins CCDC39 and CCDC40, maintains the integrity of the microtubules [8,9]. The outer doublets are connected to the central pair by radial spokes (RS) [6].

Axonemal structures are preassembled in the cytoplasm with the help of chaperones and preassembly factors such as DNAAF1, DNAAF2, DNAAF3, DNAAF4, DNAAF5, DNAAF6, DNAAF7 and DNAAF11 [10,11,12,13,14,15,16]. These structures interact together to form the 9 + 2 axonemal structure, creating a dependency on each other for the assembly of ciliary proteins. In fact, studies over the last 15 years have confirmed this, showing that a deficiency in any of the DNAAFs or outer dynein arm docking components (such as ODAD1, ODAD2, ODAD3, ODAD4 and ODAD5) leads to a deficiency in the assembly of ODAs to the axonemes [17,18,19,20,21]. Consequently, the outer dynein arm heavy chain DNAH5 is partially or completely absent from the axonemes in individuals carrying pathogenic variants not only in *DNAH5* but also in *ODAD* and *DNAAF* genes.

To date, pathogenic variants in more than 50 genes have been identified to cause PCD [22,23]. The diagnosis of PCD relies on several tools, both clinical and pre-clinical [24,25,26,27]. After a clinical examination, measurements of nasal nitric oxide (nNO) are performed, as low nNO levels correlate with PCD. Nasal brushing samples are then examined by high-speed video microscopy analysis (HSVMA) to evaluate ciliary beat frequency and by transmission electron microscopy (TEM) to investigate ultrastructural defects [6,20,24,26]. Nowadays, genetic analysis has become more accessible and affordable. To confirm the genetic analysis and address potential secondary defects and artifacts from TEM, immunofluorescence analysis (IF) of samples is also recommended [24,28].

Despite PCD being a common hereditary disease in consanguineous marriages, more investigations are needed in Turkey. Not only clinical, but also preclinical approaches for diagnosis should be carried out according to global algorithms. The exact incidence of PCD is unknown, but an increasing number of cases predicted as PCD remain underdiagnosed. Since 2013, nasal brushing samples for IF and TEM have been prepared from individuals suspected of having PCD. Although no single diagnostic tool is sufficient to detect all PCD cases, IF analysis, being less expensive and faster, has a strong chance of becoming a more popular diagnostic tool [29,30]. This study aims to evaluate IF analysis for PCD-suspected individuals and to correlate clinical and genetic findings (when available) with IF results. As there are no clinical and IF-correlated studies reported from Turkey, this study represents the first IF results from PCD-affected individuals in Turkey.

## 2. Materials and Methods

### 2.1. Study Design

All individuals suspected of having PCD who originated from Turkey were included in this cross-sectional study. A highly likely diagnosis of PCD in these individuals was determined according to the following criteria: (1) a clinical history of symptoms suggestive of PCD in combination with (2) low nasal nitric oxide, (3) abnormal ciliary beat frequency and pattern in high-speed video-microscopy analysis and/or (4) pathogenic biallelic mutations. Individuals with other chronic lung diseases with less likely diagnosis of PCD according to ERS guidelines were excluded. All participants provided informed consent.

Physical examinations of 54 individuals (31 females and 23 males) were conducted at the Hacettepe University Faculty of Medicine in the department of Pediatric Pulmonology. Infants with typical symptoms, such as nasal congestion, recurrent airway infections, and bronchiectasis, were examined using X-ray imaging. For all medical tests, including physical examinations, blood tests and further PCD diagnostic protocols, mandatory approval was obtained from the Institutional Ethics Review Board of the Hacettepe University Faculty of Medicine. Cases with low nNO were considered candidate PCD individuals and were subjected to ciliary beat frequency analysis by High-Speed Video Microscopy (HSVM). All PCD-suspected cases were diagnosed based on the ERS clinical guidelines [9].

### 2.2. Public and Patient Involvement Statement

In this research, the study was designed entirely by the scientists and the participants only provided consent without having a role in the study’s design, analysis, or dissemination. Because of this, the study would not be considered as Public and Patient Involvement research.

### 2.3. X-Ray Imaging

Routine X-ray imaging for candidate patients was performed at Hacettepe University.

### 2.4. Nasal Nitric Oxide (nNO)

Nasal NO was measured by an experienced physician at Pediatric Pulmonology Department through a NIOX-MINO (Circassia, Oxford, UK) device. All tests were made based on a flow ratio as previously described [31].

### 2.5. High-Speed Video Microscopy (HSVM)

High-speed video microscopy analysis was conducted according to previous studies [27]. Nasal brushing samples of the inferior turbinate were obtained by physicians at the Department of Pediatric Pulmonology of Hacettepe University. The nasal brushing samples were immediately immersed in RPMI medium, and a small droplet was then transferred onto a glass slide. A high-speed camera attached to an inverted microscope Leica DMI3000B (Leica Microsystems, Wetzlar, Germany) was used to capture images by using a 100× objective. For each individual, the frequency of beating and different beat patterns were recorded and noted in at least three or four non-overlapping areas. Matlab software (R2018b, MATLAB 9.5) was used to calculate the CBF (ciliary beating frequency) value (reference values were CBF 12 Hz, SD 0.8 at 37 °C).

### 2.6. Immunofluorescence Analysis

After obtaining respiratory epithelial cells by brushing the nasal epithelial area of patients (#21103, Medbar, İzmir, Turkey), the cells were suspended in RPMI 1640 medium (#01-100-1A, Biological Industries, Cromwell, CT, USA). To make a fresh, well-preserved ciliated epithelial cell smear, samples were immediately spread onto glass slides. For physical fixation, the slides were air-dried and then stored at −80 °C. For the IF procedure, samples were fixed with 4% paraformaldehyde for 15 min (RT). Then, they were permeabilized with 0.2% Triton-X for 10 min (RT) and incubated with 1% skim milk for non-specific blockage at RT overnight. These steps were followed by primary antibody incubation (3–4 h at RT) and secondary antibody incubation (30 min RT). Slides from healthy control individuals and PCD-suspected individuals were tested simultaneously with primary antibodies. Monoclonal mouse anti-DNAH5 and polyclonal rabbit anti-GAS8 (HPA041311) primary antibodies were used for double labeling at 1:500 dilution. Monoclonal mouse anti-DNAH11 and polyclonal rabbit anti-RSPH9 (HPA031703) primary antibodies were double-labeled at 1:300 dilution. Polyclonal ARMC4 antibodies were obtained from Atlas Antibodies (HPA037829; 1:100 dilution). Goat anti-mouse Alexa Fluor 488 and anti-rabbit Alexa Fluor 546 secondary antibodies were used as 1:1000 dilution. To stain the nuclei, we used Hoechst 33342 (Sigma, St. Louis, MO, USA). After mounting the slides, IF analyses were performed using a Zeiss Apotome Axiovert 200 microscope manufactured by Carl Zeiss AG (Jena, Germany) (AxioVision v.4.8 software). Presentative figures were created accordingly [20].

### 2.7. Statistical Analysis

Descriptive statistics were used to summarize clinical (e.g., age, gender, lobectomy, etc.) and laboratory findings (e.g., nasal NO levels, immunofluorescence results, high-speed video microscopy patterns). Continuous variables are presented as median (range), while categorical variables are expressed as valid percentages. ANOVA was used to compare continuous variables across motility categories, and Chi-square tests were used to assess associations between categorical variables. Crosstab analysis was conducted to evaluate relationships between motility patterns and immunofluorescence results. A significance level of *p* < 0.05 was applied to all tests, with statistical analyses performed using the Statistical Package for the Social Sciences (SPSS) version 23.0 (SPSS Inc, Chicago, IL, USA).

## 3. Results

### 3.1. Clinical Features

The study involved patients who had been clinically diagnosed as PCD-suspected individuals. Among the participants, 44.4% were male, ranging in age from 5 to 24 years. Females composed 55.6% of the population, with an age range between 12 and 27 years. All individuals included in this study, with a consanguinity rate of 79.2%, exhibited clinical symptoms of PCD, suffering from recurrent symptoms of upper and lower airway infections and chronic wet cough. Clinical findings varied among individuals. Among them, 61% suffered from sinusitis, 67.9% had a history of bronchiectasis, 40.7% had hearing defects, and 76% had a low nNO ratio. Additionally, X-ray imaging revealed that 37.7% had situs inversus. Clinical and diagnostic findings are summarized in Table 1, while clinical, laboratory and immunofluorescence labeling statistics are presented in Table 2 and Table 3.

The study included 17 individuals presenting with hearing defects, of which 14 are descendants from consanguineous marriages, 11 are associated with bronchiectasis, and 8 with situs inversus. Of the 17, 8 presented abnormal labeling of DNAH5 and only 1 individual showed abnormal labeling of DNAH11.

Five individuals had lobectomy history. One individual presented with absent labeling of DNAH5, another one with absent labeling of GAS8 and the last one with absent labeling of DNAH11.

Individual 60 (female) was a distinct case that was observed at Hacettepe Pediatric Pulmonology Clinic since infancy. She exhibited recurrent lung infections, low nNO levels and a stiff ciliary beat pattern. Nasal epithelial samples were collected in 2015. Having become an adult recently, she was diagnosed with infertility and, in current study respiratory epithelial cells, displayed DNAH11 negative staining.

### 3.2. HSVM Analysis

HSVM analysis determined that 39.6% of the individuals showed minimal residual ciliary beating, 22.9% had almost immotile ciliary movement, 12.5% had reduced amplitude, and 14.6% had stiff beating pattern. Only four samples were completely immotile, and one was recorded as hyperkinetic. Ciliary beating patterns are presented in Table 3 (the Supplementary Videos S1–S10 represent the ciliary beating patterns from unaffected control and individuals with *DNAH5*, *ARMC4* mutations and suspected PCD).

### 3.3. Genetic Results

Genetic results were available for eight individuals. We identified individuals 33, 119 and 30 with homozygous variants in *DNAH5*, individuals 9, 107 and 109 with homozygous variants in *DNAAF1*, individual 19 with homozygous *ARMC4* missense variants and individual 32 with homozygous variants in *CCDC40*.

Individual 33, a descendant of a consanguineous family, presenting with situs inversus totalis and bronchiectasis, and carried biallelic missense variants in *DNAH5* (c.7615T>C; p.Trp2539Arg). Individuals 119 and 30 carried biallelic nonsense variants in *DNAH5,* respectively: c.5747G>A; p.Trp1916Ter and c.2710G>T; p.Glu904Ter. Individual 119 is also a descendant of a consanguineous family, presenting with bronchiectasis, while individual 30 did not present any consanguinity nor laterality defects. Respiratory cilia of individual 30 were almost immotile, whereas respiratory cilia of individuals 33 and 119 displayed minimal residual ciliary movements.

Individual 9, a descendant of a consanguineous family, presenting with situs inversus totalis, carried biallelic missense variants in *DNAAF1* (c.1385A>C; p.Gln462Pro) and had respiratory epithelial cells displaying a minimal residual ciliary movement. Individual 107, a descendant of a consanguineous family, presented with situs inversus totalis, carried biallelic frameshift variants in *DNAAF1* (c.1349dupC; p.Pro451fs*6) and had respiratory epithelial cells displaying hypokinetic ciliary movement. Individual 109, a descendant of a consanguineous family, presented with situs inversus totalis, carried biallelic frameshift variants in *DNAAF1* (c.1228_1232delCCAGA; p.Pro410fs*8) and had respiratory epithelial cells that were were almost immotile.

Individual 19 carried biallelic *ARMC4* missense variants (c. 2780T>G, p. Leu927Trp) and had respiratory epithelial cells displaying minimal residual ciliary movement with a reduced beating pattern.

Individual 32 carried biallelic *CCDC40* nonsense variants (c.1315C>T; p.Gln439*), is a descendant of consanguineous family, and had hypokinetic ciliary movements.

### 3.4. Immunofluorescence Analysis for Genetically Analyzed Individuals

**Figure 1:**
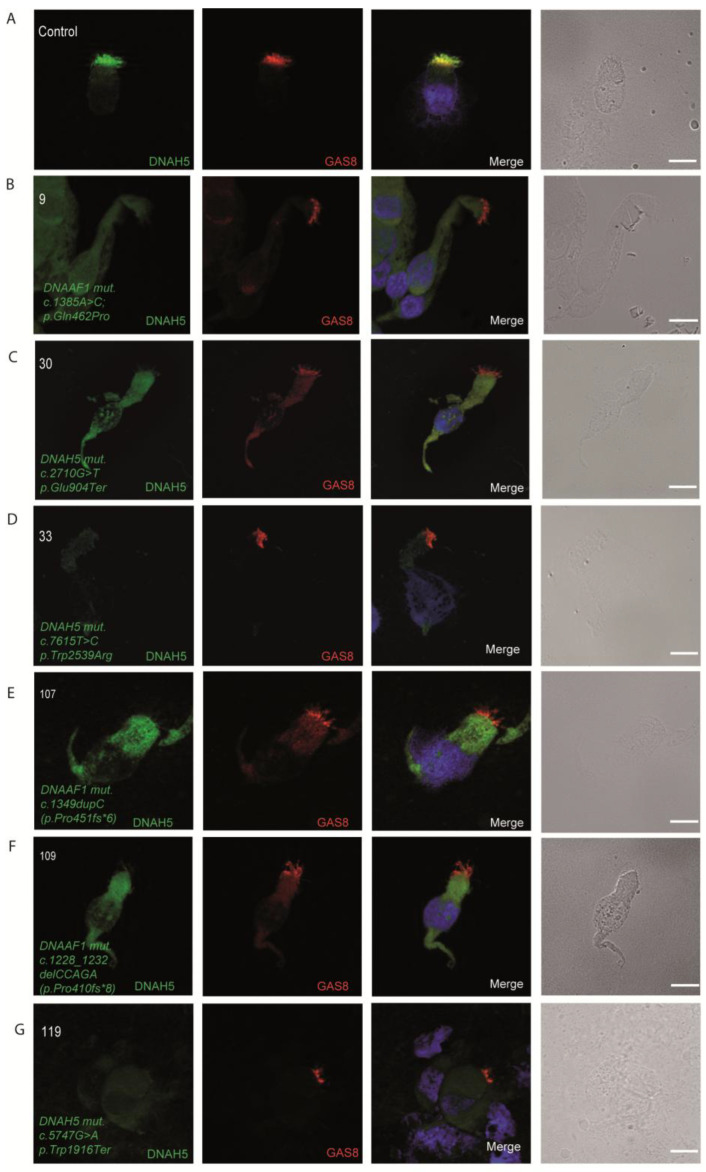
DNAH5 is undetectable in respiratory ciliary axonemes of PCD-affected individuals with pathogenic variants in *DNAH5* and *DNAAF1*. (**A**) Respiratory cilia double-labeled with antibodies directed against DNAH5 (green) and GAS8 (red) show colocalization of DNAH5 with GAS8 along the cilia from healthy controls (yellow color). (**B**–**G**) In *DNAH5-* and *DNAAF1-mutant* cells, DNAH5 is absent from the ciliary axonemes. Nuclei were stained with Hoechst 33342 (blue). Scale bars represent 10 mm.

**Figure 2:**
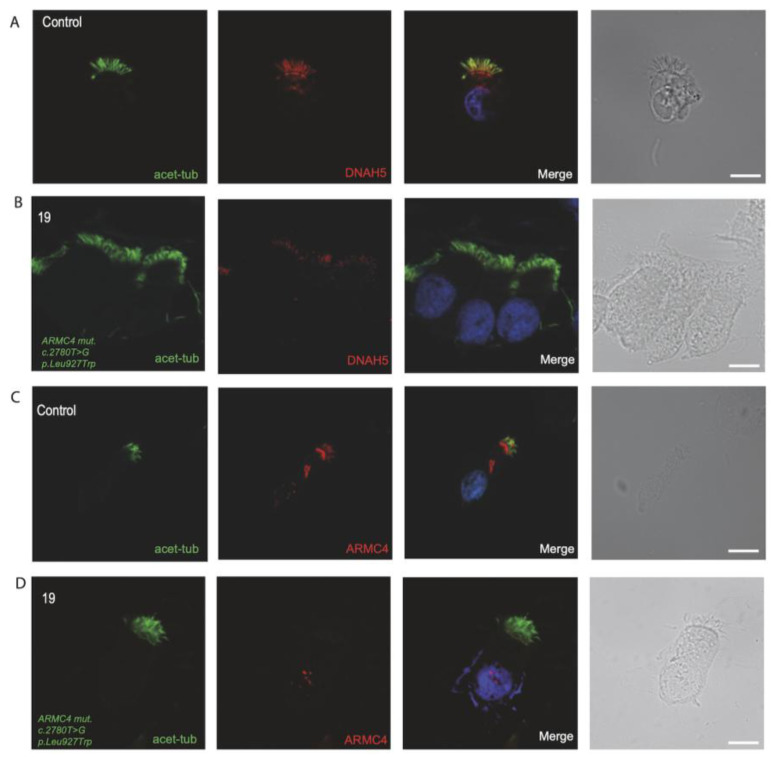
Defects in the assembly of ARMC4 and DNAH5 in respiratory ciliary axonemes of PCD-affected individual with pathogenic variants in *ARMC4*. (**A**) Respiratory cilia double-labeled with antibodies directed against acetylated tubulin alpha (green) and DNAH5 (red) show colocalization of acetylated tubulin with DNAH5 along the cilia from healthy controls (yellow color). (**B**) In contrast, proximal localization of DNAH5 is shown in *ARMC4* mutant axonemes (**C**). Respiratory cilia double-labeled with antibodies directed against acetylated alpha tubulin (green) and ARMC4 (red) show colocalization of acetylated tubulin with ARMC4 on the cilia from unaffected controls. (**D**) In *ARMC4*-mutant cells, ARMC4 is absent from the ciliary axonemes. Nuclei were stained with Hoechst 33342 (blue). Scale bars represent 10 mm.

**Figure 3:**
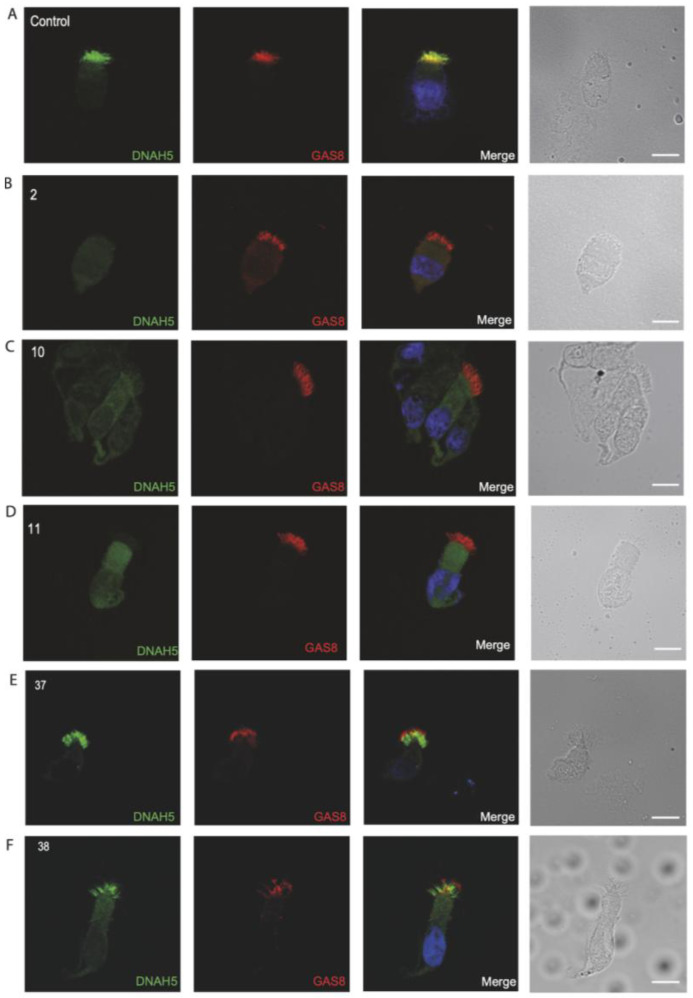
Immunofluorescence analysis of PCD-suspected individuals reveals the absence or abnormal localization of DNAH5 in the respiratory ciliary axonemes. (**A**) Respiratory cilia double-labeled with antibodies directed against DNAH5 (green) and GAS8 (red) show colocalization of DNAH5 with GAS8 along the cilia from healthy controls (yellow color). (**B**–**D**) In contrast, DNAH5 is absent in respiratory axonemes of PCD-suspected individuals. (**E**,**F**) Two siblings, showing proximal staining pattern of DNAH5 in the ciliary axonemes of respiratory epithelium. Nuclei were stained with Hoechst 33342 (blue). Scale bars represent 10 mm.

**Figure 4:**
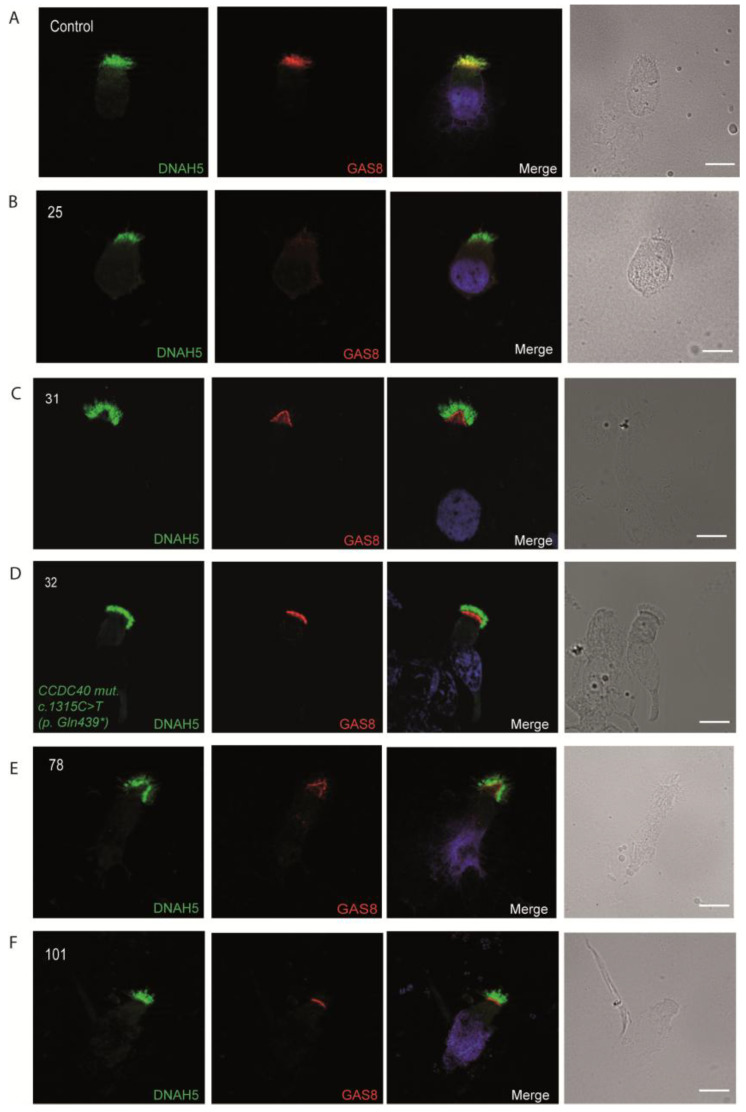
Immunofluorescence analysis of PCD-suspected individuals reveals an absence of GAS8 from the respiratory ciliary axonemes. (**A**) Respiratory cilia double-labeled with antibodies directed against DNAH5 (green) and GAS8 (red) show colocalization of DNAH5 with GAS8 along the cilia from unaffected controls (yellow color). (**B**,**E**,**F**) In contrast, GAS8 is absent in respiratory axonemes of PCD-suspected individuals. (**C**,**D**) Two siblings, showing absence of GAS8 in the ciliary axonemes of respiratory epithelium. (**D**) *CCDC40 mutant* respiratory cells were negative for GAS8. Nuclei were stained with Hoechst 33342 (blue). Scale bars represent 10 mm. * stands for termination of translation of the protein.

To confirm the pathogenicity of the identified variants, we analyzed the localization of DNAH5 in the respiratory epithelial cells of the seven individuals identified by IF microscopy. DNAH5 was undetectable in individuals 33, 119, 30, 9, 107 and 109 (shown in Figure 1) and localized only to the proximal axonemes in individual 19 (shown in Figure 2). We also analyzed the localization of ARMC4 in the respiratory epithelial cells of individual 19, and we could not detect any signal, confirming the absence of the protein from the ciliary axonemes (shown in Figure 2). DNAH5 was also abnormal in individuals without genetic analysis (shown in Figure 3). In individual 32, we also did not detect GAS8 signaling by IF (shown in Figure 4). The staining results correlated with the genetic findings.

### 3.5. Diagnostic IF Analysis for Non-Genetically Analyzed Individuals

As the genetic analysis of the remaining individuals was not available and given the efficiency of IF analysis as a diagnostic tool, we performed IF analysis with different antibodies to reveal the ciliary defect at the molecular level. We used antibodies targeting DNAH5 and DNAH11 (dynein heavy chains of the outer dynein arm complex), GAS8 (component of the nexin–dynein regulatory complex n-DRC) and RSPH9 (component of the radial spoke complex).

As mentioned above, the localization of DNAH5 is affected in individuals with pathogenic variants in *DNAH5*, other ODA structural components, ODA docking-associated genes (*ODADs*) and preassembly factors (*DNAAFs*). We also targeted the localization of DNAH11 as well, as this component of the outer dynein arm is affected only in individuals with pathogenic variants in *DNAH11*, *CFAP45* and *CFAP52* [32]. Furthermore, we analyzed the localization of GAS8, which not only indicates a deficiency in GAS8 but also in the ruler proteins CCDC39 and CCDC40 [9]. Additionally, we targeted the localization of RSPH9, as this radial spoke component is absent in individuals with pathogenic variants in *RSPH9*, *RSPH4A* and other radial spoke genes. Although the four antibodies cover a wide range of genetic defects related to PCD, they do not cover CP, isolated IDA and other N-DRC and RSP defects, for example. Therefore, a normal IF does not exclude PCD in these cases.

**Figure 5:**
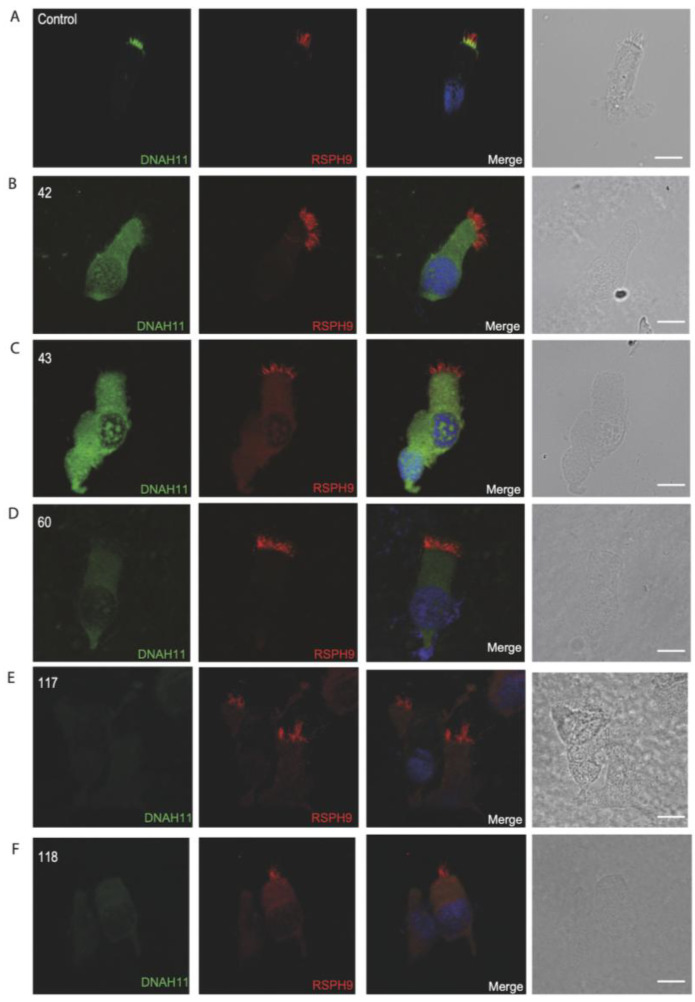
Immunofluorescence analysis of PCD-suspected individuals reveals the absence of DNAH11 from the respiratory ciliary axonemes. (**A**) Respiratory cilia double-labeled with antibodies directed against DNAH11 (green) and RSPH9 (red) show colocalization of DNAH11 with RSPH9 in the proximal ciliary axonemes from unaffected controls (yellow color). (**B**–**D**) In contrast, DNAH11 is absent in respiratory axonemes of PCD-suspected individuals. (**E**,**F**) Two siblings, showing absence of DNAH11 in the ciliary axonemes of respiratory epithelium. Nuclei were stained with Hoechst 33342 (blue). Scale bars represent 10 mm.

**Figure 6:**
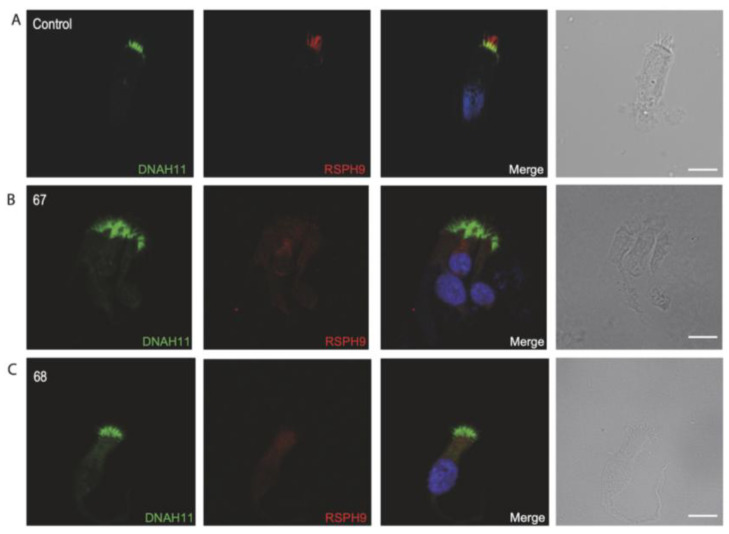
Immunofluorescence analysis of PCD-suspected individuals reveals the absence of RSPH9 from the respiratory ciliary axonemes. (**A**) Respiratory cilia double-labeled with antibodies directed against DNAH11 (green) and RSPH9 (red) show a proximal localization of DNAH11 with RSPH9 along the cilia from unaffected controls (yellow color). (**B**,**C**) Siblings, showing RSPH9 is absent in respiratory axonemes of PCD-suspected individuals. Nuclei were stained with Hoechst 33342 (blue). Scale bars represent 10 mm.

Repeated IF analysis revealed an absent localization of DNAH5 in 16 individuals, an absent localization of GAS8 in 7 individuals, an absent localization of DNAH11 in 5 individuals and an absent localization of RSPH9 in 1 individual, pointing to the possible PCD defect in these cases (shown in Figure 3, Figure 4, Figure 5 and Figure 6). To corroborate the negative staining observed in some individuals, we analyzed affected siblings whenever available. Consequently, siblings 37 and 38 were DNAH5-negative (proximal), siblings 31 and 32 were negative for GAS8, siblings 117 and 118 were DNAH11-negative, and siblings 67 and 68 were negative for RSPH9 (shown in Figure 3, Figure 4, Figure 5 and Figure 6) (See also Supplementary Figures S1 and S2, providing additional information for the remaining DNAH5 and GAS8 abnormal immunofluorescence analysis).

### 3.6. Proposed Diagnostic Algorithm for PCD

To evaluate the performance of immunofluorescence (IF) as a diagnostic tool for PCD, we analyzed individuals in two groups: Group A: Individuals with available genetic analysis and Group B: Individuals without genetic analysis. We calculated the sensitivity and specificity of IF compared to genetic analysis using a 2 × 2 contingency table (Table 4):

In group A (n = 8, available genetic analysis), IF correlated with genetic results in 8 cases (True Positives, TP = 8). IF was absent in 0 genetically confirmed cases (False Negative, FN = 0). In group B (n = 46, without genetic analysis), IF was absent in 17 of 46 individuals (True Negatives, TN = 17), and IF was present in 29 of 46 individuals (False Positives, FP = 29). Sensitivity and specificity calculations were made depending on the formula: Sensitivity = TP/(TP + FN) × 100 = 8/(8 + 0) × 100 = 100% and Specificity = TN/(TN + FP) × 100 = 17/(29 + 17) × 100 = 17/46 ≈ 37%.

**Figure 7:**
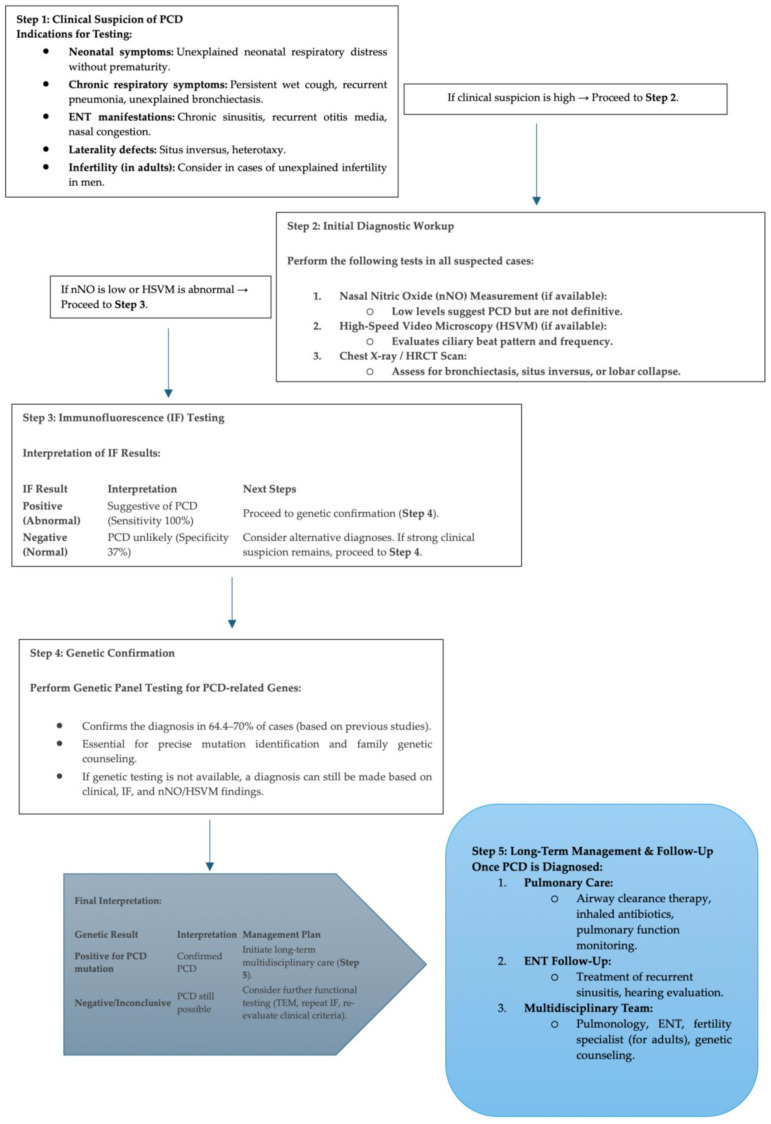
PCD diagnostic algorithm flow.

IF correctly identified 100% of PCD cases when genetic analysis confirmed the diagnosis and correctly excluded 37% of non-PCD cases. An algorithm for PCD diagnosis is formed (shown in Figure 7).

## 4. Discussion

Despite the identification of more than 50 genes associated with PCD, more than 30% of PCD cases remain genetically unresolved [29,33]. Recently, our colleagues published a survey on PCD cases identified in Turkey, both clinically and genetically [31]. However, the approach to these cases is complex and includes not only clinical diagnostics but also laboratory techniques like IF and TEM analyses [17,34]. In our study, we aimed to provide insights using clinical approaches guided by global algorithms, employing IF on Turkey-based cases.

Here, we present the first clinical PCD cases from Turkey with IF correlation in a large patient group. As a result, PCD candidate individuals with symptoms such as persistent cough, purulent expectoration, and rhinorrhea were identified [6,24]. Although results varied, bronchiectasis was the most prevalent clinical finding in our patients, which was consistent with the meta-analysis performed by Goutaki et al. [35].

A global perspective on PCD diagnosis has emerged through cohort studies conducted across diverse populations. In 2022, Hannah et al. conducted a large cohort study, comparing the prevalence of PCD across different ethnic groups. Since most PCD genetic studies have been performed in European and North American populations, their research aimed to provide a more global perspective. Their study suggested that including a worldwide cohort study could highlight the genetic variations between different ethnic groups [36]. Another cohort study performed in China included 26 PCD patients and identified *DNAH5* as the most prevalent mutation among genetically diagnosed individuals. Through clinical assessments and whole-genome sequencing (WGS), they demonstrated that clinical features aligned with genetic findings [37]. In the North American population, PCD studies have been relatively limited. However, Poplawska et al. investigated PCD cases and highlighted the significance of genetic testing in the PCD area, particularly while socioeconomic factors were thought to be the cause of recurrent respiratory infections [38].

Our study adds to this growing body of evidence by providing the first clinical and IF-based insights into PCD cases from Turkey. The high prevalence of *DNAH5* mutations in our cohort aligns with findings from European populations and a recent Chinese study, suggesting a shared genetic basis. However, the identification of unique clinical presentations and genetic variants in our patients highlights the importance of expanding PCD research to diverse populations.

Furthermore, an international survey, as a part of the COVID-PCD cohort, including participants from Europe, North America and other non-European countries, was conducted [39]. This questionnaire study revealed significant variations in diagnostic testing for PCD between countries. While most of participants were tested, not all underwent all three major tests (nNO, biopsy and genetics). The study underlined that genetic testing is more common now than in previous surveys, but also highlighted the need for more complete diagnostics, especially for people diagnosed long ago or with situs abnormalities. Similarly, Pedersen et al. emphasized the importance of correlating genetic findings with clinical outcomes to improve diagnostic accuracy [40]. Our study reinforces these conclusions, demonstrating that IF serves as a reliable initial screening tool, particularly in resource-limited settings where genetic testing is not always accessible.

This study was limited by having only genomic findings from eight individuals with genetic variants. One of the key strengths of our study is its demonstration of high sensitivity (100%) of IF for detecting PCD in genetically confirmed cases. However, its moderate specificity (37%) highlights the need for confirmatory genetic testing in IF-positive individuals to reduce false positives.

Despite this limitation, IF remains a valuable diagnostic tool, particularly in clinically suspected individuals. It is widely accessible and cost-effective, and provides rapid results compared to genetic testing and transmission electron microscopy.

The relatively lower specificity observed in this study may reflect the complexity of diagnosing PCD, as multiple ciliary protein abnormalities contribute to the disease, and secondary ciliary defects can influence the IF pattern. Although this study’s IF panel covered key PCD-related proteins, more than 50 genes and their related proteins are linked to PCD [22]. Even so, previous studies have shown that when interpreted within the appropriate clinical context, IF remains a reliable and feasible method for initial screening and diagnosis [25,28,41].

Nevertheless, IF analysis in individuals with *DNAH5* and *DNAAF1* variants (patients 33, 119, 30 and 9, 107, 109) correlated with the genetic results. Moreover, HSVM analysis showed that these individuals displayed a range from minimal residual to almost immotile ciliary beating, as previously reported in individuals with pathogenic variants in *DNAH5* and *DNAAF1* [10,13].

Consanguinity in individuals with *DNAH5* pathogenic variants and negative immunofluorescence labeling was consistent with the literature [42].

Respiratory cells from individual 19 with homozygous *ARMC4* missense variants (c. 2780T>G, p. Leu927Trp) displayed a proximal staining pattern of DNAH5 and complete absence of ARMC4, consistent with previous publications [18]. HSVM analysis in the cilia of this individual revealed a minimal residual beating pattern with reduced amplitude, consistent with the findings of Raidt et al. [27].

Respiratory cells from siblings 37 and 38 showed a proximal localization of DNAH5 by IF, and both had immotile cilia. The male sibling did not have bronchiectasis or situs inversus, but the female sibling did, indicating that DNAH5 deficiency had different effects on the two siblings [41,43]. Both siblings showed an almost immotile cilia beat pattern via HSVM, consistent with previous studies [27,41].

Respiratory cells from siblings 31 and 32 showed an absence of GAS8 from the ciliary axonemes, consistent with CCDC40 deficiency. They shared similar clinical symptoms, and their parents were consanguineous. The remaining GAS8-absent individuals had mostly consanguinity-positive backgrounds (with no genetic data), supporting the literature [44]. We observed that the cilia of these siblings had reduced amplitude, in line with the fact that GAS8 abnormalities cause reduced or disorganized ciliary beating [45]. The remaining individuals with abnormal GAS8 staining exhibited a minimal residual and reduced beating pattern, except for one individual recorded as having minimal residual and almost immotile beating pattern.

Respiratory cells from siblings 117 and 118 showed an absence of DNAH11. The male sibling had a history of lobectomy. Both siblings exhibited consanguinity and bronchiectasis but showed no concerns with heterotaxy. The beating pattern of their cilia was stiff, correlating with the previous studies [27,46] that reported increased ciliary beating frequency in patients with known *DNAH11* mutations. Other individuals with DNAH11-negative staining included two individuals with hearing loss; HSVM analysis in one of them showed a stiff beating pattern, aligning with the previous studies [27,46,47]. Another female patient with DNAH11 negative staining had fertility issues. Although DNAH11 abnormality was associated with male infertility recently [48], a woman with primary infertility has been reported to carry homozygous pathogenic variants in *DNAH11* [49]. Additionally, HSVM revealed that the stiff beating pattern was present at the distal part of the cilia, and the cilia were likely shorter in this individual. These findings aligned with the study by Nussbaumer et al. [47]. The remaining individuals with DNAH11 abnormality exhibited stiff ciliary beating patterns. Schreck et al. examined fertility outcomes in male and female PCD diagnosed patients from the U.S.A., Canada, European countries, the U.K. and some other countries. The study included clinical features and some available genetic results. Among women with known genetic results, those with infertility had *DNAH5*, *DNAAF3*, *ZMYND10*, *CCDC40*, *RSPH9* and *HYDIN* mutations [50]. However, no immunofluorescence labeling was presented. In our cohort, the female patient with DNAH11-negative staining with infertility issues, consistent with previous reports of primary infertility in individuals with *DNAH11* mutations [49]. These findings highlight the broader clinical implications of PCD and the need for comprehensive genetic, clinical and IF evaluations, particularly when TEM or WES is not feasible.

Respiratory cells from siblings 67 and 68, descendants of a consanguineous marriage and born deaf, tested negative for RSPH9 by IF analyses. Interestingly, these individuals did not display any laterality defects, consistent with the observation that individuals with radial spoke defects typically present with situs solitus [51]. Although the genetic background of these siblings remained unknown throughout our investigation, PCD could be indicated due to the IF results and clinical phenotype [52]. It has been noted that some rotational or metronome-like (stiff) movements can be seen in patients with radial spoke abnormalities on HSVM; our study revealed that these siblings had stiff ciliary beating [53].

Moreover, the majority of the remaining individuals had impaired mucociliary clearance and bronchiectasis due to minimal residual or immotile cilia, and most had abnormal DNAH5 immunostaining (either negative or proximal staining pattern). Consistently, aberrant DNAH5 localization found by IF in individuals correlated with immotile or minimal residual ciliary beating found by HSVM, indicating that our findings were in line with previous studies [41].

*DNAH5* is recognized as the gene with the most prevalent mutations in European families, as previously reported [54]. We discovered that 50% of the patients had abnormal DNAH5 staining results and, consequently, defects in the outer dynein arms—the ciliary stroke power generators. ODA defects can be easily detected under an electron microscope [55]. Indeed, we demonstrated here that individuals with homozygous *DNAH5* pathogenic variants could be identified by IF labeling, verifying their clinical PCD symptoms.

A key limitation of this study is the absence of ultrastructural analysis using transmission electron microscopy (TEM), which is traditionally considered a gold standard for diagnosing PCD-related ciliary defects. While our study effectively utilized IF and genetic testing, TEM provides direct visualization of ciliary ultrastructural defects, aiding in the confirmation of PCD cases, particularly in genetically unresolved individuals. TEM was not included in this study due to lack of availability, cost constraints, and limited accessibility in our clinical setting. Given the requirement for specialized expertise and sample preparation challenges, TEM is often not feasible in all diagnostic centers. Despite the absence of TEM, IF demonstrated high sensitivity (100%) and provided molecular insights into ciliary defects, complementing genetic diagnostics.

## 5. Conclusions

The study identified a correlation between IF and genetics in genetically analyzed individuals and a correlation between IF and clinically suspected PCD individuals. According to our data, the most frequent defect identified in clinically suspected PCD candidates in our group is related to the outer dynein arms (via the absence of DNAH5). We showed that immunofluorescence, compared to TEM, has significant potential to become a more common diagnostic strategy by being less expensive and less time-consuming. Overall, even in the absence of genetic data or electron microscopy, immunofluorescence remains a potent tool for PCD diagnosis.

## Figures and Tables

**Table 1:** Clinical, genetic and demographic findings of PCD-affected individuals.

Individual ID	Age	Sex	Symptoms	Bronchiectasis	Situs Inversus	Consanguinity	Genetic Variant	HSVM	DNAH5	GAS8	DNAH11	RSPH9
2	19 y	F	Recurrent Lung Infection, Hearing Defect	yes	yes	yes		NA	negative	normal	normal	normal
6	11 y	M	Recurrent Lung Infection, Low Nasal NO, Sinusitis, Hearing Defect	no	no	no		Minimal residue movement	normal	normal	normal	normal
9	19 y	M	Recurrent Lung Infection, Low Nasal NO, Sinusitis	no	yes	yes	*DNAAF1* hom: c.1385A>C; p.Gln462Pro	Minimal residue movement	negative	normal	normal	normal
10	15 y	F	Recurrent Lung Infection, Low Nasal NO, Sinusitis, Hearing Defect	yes	no	yes		Minimal residue movement	negative	normal	normal	normal
11	24 y	F	Recurrent Lung Infection, Low Nasal NO, Sinusitis, Lobectomy	yes	no	yes		Immotile cilia	negative	normal	normal	normal
12	24 y	M	Recurrent Lung Infection	yes	no	no		Minimal residue movement	normal	normal	normal	normal
14	15 y	F	Recurrent Lung Infection, Hearing Defect	no	yes	yes		Almost immotile cilia	negative	normal	normal	normal
15	12 y	F	Recurrent Lung Infection, Low Nasal NO, Sinusitis	no	no	yes		Minimal residue movement	negative	normal	normal	normal
17	13 y	F	Recurrent Lung Infection, Low Nasal NO	yes	no	no		Immotile cilia	negative	normal	normal	normal
18	17 y	F	Recurrent Lung Infection	no	yes	yes		Almost immotile cilia	normal	normal	normal	normal
19	22 y	F	Recurrent Lung Infection, Hearing Defect	no	yes	yes	*ARMC4* hom: c.2780T>; p.Leu927Trp	Minimal residue movement and reduced amplitude	proximal	normal	normal	normal
20	20 y	F	Recurrent Lung Infection	no	no	yes		Almost immotile cilia	normal	normal	normal	normal
21	22 y	F	Recurrent Lung Infection, Low Nasal NO, Sinusitis, Hearing Defect	yes	no	yes		Minimal residue movement	normal	normal	normal	normal
24	20 y	F	Recurrent Lung Infection, Low Nasal NO	yes	no	yes		Almost immotile cilia	negative	normal	normal	normal
25	10 y	M	Recurrent Lung Infection, Low Nasal NO, Sinusitis	yes	yes	yes		Minimal residue movement	normal	negative	normal	normal
28	13 y	M	Recurrent Lung Infection, Low Nasal NO	yes	no	yes		Reduced amplitude	normal	negative	normal	normal
29	20 y	M	Recurrent Lung Infection, Low Nasal NO, Hearing Defect	yes	yes	no		Minimal residue movement	normal	negative	normal	normal
30	15 y	M	Recurrent Lung Infection, Low Nasal NO	yes	no	no	*DNAH5* hom: c.2710G>; p.Glu904Ter	Almost immotile cilia	negative	normal	normal	normal
31	22 y	F	Recurrent Lung Infection, Low Nasal NO, Sinusitis	yes	no	yes		Reduced amplitude	normal	negative	normal	normal
32	8 y	M	Recurrent Lung Infection	no	no	yes	*CCDC40* hom: c.1315C>T; p. Gln439Ter	Reduced amplitude	normal	negative	normal	normal
33	21 y	F	Recurrent Lung Infection, Low Nasal NO, Sinusitis	yes	yes	yes	*DNAH5* hom: c.7615T>; p.Trp2539Arg	Minimal residue movement	negative	normal	normal	normal
36	15 y	F	Recurrent Lung Infection, Low Nasal NO	yes	no	no		Hyperkinetic cilia	normal	normal	normal	normal
37	22 y	M	Recurrent Lung Infection, Low Nasal NO, Sinusitis	no	no	yes		Almost immotile cilia	proximal	normal	normal	normal
38	16 y	F	Recurrent Lung Infection, Low Nasal NO, Sinusitis	yes	yes	yes		Almost immotile cilia	proximal	normal	normal	normal
42	14 y	F	Low Nasal NO	no	yes	yes		Stiff pattern	normal	normal	negative	normal
43	24 y	F	Low Nasal NO, Recurrent Lung Infection, Hearing Defect	no	no	no		NA	normal	normal	negative	normal
44	16 y	F	Recurrent Lung Infection, Low Nasal NO, Sinusitis, Hearing Defect	yes	no	yes		Minimal residue movement	negative	normal	normal	normal
46	16 y	M	Recurrent Lung Infection, Low Nasal NO, Sinusitis, Hearing Defect	yes	no	yes		Minimal residue movement	proximal	normal	normal	normal
48	14 y	M	Recurrent Lung Infection, Low Nasal NO, Sinusitis	yes	no	yes		Reduced amplitude	normal	negative	normal	normal
51	13 y	M	Recurrent Lung Infection, Sinusitis, Hearing Defect	yes	no	yes		Almost immotile cilia	normal	normal	normal	normal
53	20 y	F	Recurrent Lung Infection, Low Nasal NO, Sinusitis, Lobectomy	yes	no	no		NA	normal	normal	normal	normal
60	24 y	F	Recurrent lung infection, Low Nasal NO, Infertility	no	no	no		Stiff pattern	normal	normal	negative	normal
63	5 y	M	Recurrent Lung Infection, Hearing Loss, Low Nasal NO	no	no	yes		Stiff pattern	normal	normal	normal	normal
66	21 y	M	Recurrent Lung Infection, Low Nasal NO, Sinusitis	yes	no	yes		Minimal residue movement	proximal	normal	normal	normal
67	20 y	F	Recurrent Lung Infection, Low Nasal NO, Sinusitis, Hearing Defect	yes	no	yes		Stiff pattern	normal	normal	normal	negative
68	17 y	M	Recurrent Lung Infection, Low Nasal NO, Sinusitis, Hearing Defect, Lobectomy	yes	no	yes		Stiff pattern	normal	normal	normal	negative
72	14 y	M	Recurrent Lung Infection, Low Nasal NO	yes	yes	no		Minimal residue movement	normal	normal	normal	normal
73	21 y	M	Recurrent Lung Infection, Low Nasal NO, Sinusitis, Hearing Defect	yes	no	yes		Minimal residue movement	normal	normal	normal	normal
76	16 y	M	Recurrent Lung Infection, Sinusitis, Hearing Defect	yes	no	yes		Minimal residue movement	negative	normal	normal	normal
77	19 y	M	Recurrent Lung Infection, Low Nasal NO	yes	no	yes		NA	normal	normal	normal	normal
78	12 y	M	Recurrent Lung Infection, Low Nasal NO, Sinusitis	no	yes	yes		Minimal residue movement	normal	negative	normal	normal
83	15 y	F	Recurrent Lung Infection, Low Nasal NO, Sinusitis, Hearing Defect	no	yes	yes		NA	normal	normal	normal	normal
96	22 y	M	Recurrent Lung Infection, Low Nasal NO, Sinusitis, Hearing Defect	yes	no	yes		Minimal residue movement	negative	normal	normal	normal
100	12 y	F	Recurrent Lung Infection, Low Nasal NO	no	yes	yes		Almost immotile cilia	normal	normal	normal	normal
101	23 y	F	Recurrent Lung Infection, Low Nasal NO, Sinusitis, Lobectomy	yes	no	yes		Reduced amplitude	normal	negative	normal	normal
102	24 y	F	Recurrent Lung Infection, Hearing Defect	yes	yes	yes		NA	normal	normal	normal	normal
107	17 y	F	Recurrent Lung Infection, Hearing Defect, Sinusitis	yes	yes	yes	*DNAAF1* hom: c.1349dupC; p.Pro451fs Ter6	Minimal residue movement	negative	normal	normal	normal
109	27 y	F	Recurrent Lung Infection, Hearing Defect, Sinusitis	no	yes	yes	*DNAAF1* hom: c.1228_1232delCCAGA; p.Pro410fs Ter8	Almost immotile cilia	negative	normal	normal	normal
110	22 y	F	Recurrent Lung Infection, Sinusitis	yes	yes	yes		Almost immotile cilia	negative	normal	normal	normal
117	14 y	F	Recurrent Lung Infection, Low Nasal NO, Sinusitis	yes	no	yes		Stiff pattern	normal	normal	negative	normal
118	17 y	M	Recurrent Lung Infection, Low Nasal NO, Sinusitis, Lobectomy	yes	no	yes		Stiff pattern	normal	normal	negative	normal
119	18 y	F	Recurrent Lung Infection, Low Nasal NO, Sinusitis, Hearing Defect	yes	yes	no	*DNAH5* hom: c.5747G>A; p.Trp1916Ter	Minimal residue movement	negative	normal	normal	normal
#PH 1	7 y	M	Recurrent Lung Infection, Low Nasal NO, Sinusitis	NA	NA	NA		Minimal residue movement	normal	normal	normal	normal
#PH 2	12 y	F	Recurrent Lung Infection, Low Nasal NO, Sinusitis	yes	yes	yes		Minimal residue movement	normal	normal	normal	normal

y: years; M: male; F: female; NO: nitric oxide; hom: homozygous; NA: not available.

**Table 2:** Clinical and immunofluorescence labeling statistics ^1^.

Clinical Statistics Gender	N Median		Valid Percent	DNAH5 Abnormality n (% Within Group)	GAS8 Abnormality n (% Within Group)	DNAH11 Abnormality n (% Within Group)	RSPH9 Abnormality n (% Within Group)	*p* Value
Female	23	1.4444	55.6 44.4	14 (63.6%) 8 (36.4%)	2 (25.0%) 6 (75.0%)	3 (60.0%) 2 (40.0%)	1 (50.0%) 1 (50.0%)	0.432
Male	31							
Age, median (min-max)	17 (5–32)							
Consanguinity, n	41		79.2	86.4%	87.5%	60%	100%	0.409
Sinusitis, n	33		61.1	72.7%	62.5%	20%	100%	0.121
Bronchiectasis, n	36		67.9	72.7%	75%	20%	100%	0.144
Recurrent lung infection, n	53		98.1	100%	100%	80%	100%	-
Hearing defect, n	22		40.7	50%	12.5%	40%	100%	0.105
Situs inversus totalis, n	20		37.7	45.5%	37.5%	20%	0%	0.529
Lobectomy, n	5		9.3	4.5%	12.5%	20%	50%	0.438
Infertility, n	1		1.9	0%	0%	20%	0%	-

N (n): Number of patients; DNAH5: Dynein Axonemal Heavy Chain 5; GAS8: Nexin–Dynein Regulatory Complex Component; DNAH11: Dynein Axonemal Heavy Chain 11; RSPH9: Radial Spoke Head Component 9. ^1^ *p*-Value < 0.05 is considered statistically significant.

**Table 3:** Laboratory statistics.

Lab Statistics		n	Median (Min–Max)	Valid Percent
Nasal NO (ppm)		43	9 (5–16)	
High-speed video microscopy	minimal residual	22		39.6
	hyperkinetic	1		2.1
	immotile	4		8.3
	almost immotile	10		22.9
	reduced	6		12.5
	stiff	7		14.6
Immunofluorescence labeling	Normal	17		9.2
	DNAH5 abnormal	22 *		11.8
	GAS8 abnormal	8		4.3
	DNAH11 abnormal	5		2.7
	RSPH9 abnormal	2		1
	ARMC4 abnormal	1 *		0.5

ARMC4: outer dynein arm docking complex component. * Individual 19 had both DNAH5 and ARMC4 negative immunofluorescence labeling. Valid percentages represent the proportion of individuals with each ciliary motion pattern among those assessed, excluding missing data.

**Table 4:** Sensitivity and Specificity Contingency table.

	Genetic PCD (+)	Genetic PCD (−)	Total
**IF-Positive (TP)**	8	17 (FP)	25
**IF-Negative (FN)**	0	29 (TN)	29
**Total**	8	46	54

## Data Availability

The data generated and/or analyzed during the current study are available from the corresponding author upon reasonable request.

## Supplementary Materials

The following supporting information can be downloaded at: https://www.mdpi.com/article/10.3390/jcm14061941/s1, Figure S1. Immunofluorescence analysis of PCD-suspected individuals reveals the absence or abnormal localization of DNAH5 in the respiratory ciliary axonemes. (A) Respiratory cilia double-labeled with antibodies directed against DNAH5 (green) and GAS8 (red) show colocalization of DNAH5 with GAS8 along the cilia from healthy controls. (B–G,J,K) In contrast, DNAH5 is absent in respiratory axonemes of PCD-suspected individuals. (H,I) Two PCD-suspected individuals, showing proximal staining pattern of DNAH5 in the ciliary axonemes of respiratory epithelium. Nuclei were stained with Hoechst 33342 (blue). Scale bars represent 10 mm.; Figure S2. Immunofluorescence analysis of PCD-suspected individuals reveals an absence of GAS8 from the respiratory ciliary axonemes. (A) Respiratory cilia double-labeled with antibodies directed against DNAH5 (green) and GAS8 (red) show colocalization of DNAH5 with GAS8 along the cilia from unaffected controls. (B–D) In contrast, GAS8 is absent in respiratory axonemes of PCD-suspected individuals. Nuclei were stained with Hoechst 33342 (blue). Scale bars represent 10 mm.; Video S1. Control HSVM video of unaffected control; Video S2. *DNAH5 mutant* (c.2710G>T; p.Glu904Ter) Almost immotile ciliary beat of individual 30.; Video S3. *DNAH5 mutant* (c.7615T>C;p.Trp2539Arg) Minimal residual ciliary beat of individual 33.; Video S4. *DNAH5 mutant* (c.5747G>A; p.Trp1916Ter) Minimal residual ciliary beat of individual 119.; Video S5. *ARMC4 mutant* (c.2780T>G; p.Leu927Trp) Minimal residual and reduced amplitude ciliary beat of individual 19.; Video S6. Almost immotile cilia of PCD-suspected individual 38.; Video S7. Almost immotile cilia of individual 24.; Video S8. Hyperkinetic ciliary beat pattern of individual 36.; Video S9. Minimal residual and almost immotile cilia of individual 25.; Video S10. Stiff ciliary beat of individual 63.

## References

[1] Mirra V., Werner C., Santamaria F. (2017). Primary Ciliary Dyskinesia: An Update on Clinical Aspects, Genetics, Diagnosis, and Future Treatment Strategies. Front. Pediatr..

[2] Horani A., Ferkol T.W. (2018). Advances in the Genetics of Primary Ciliary Dyskinesia. Chest.

[3] Reilly M.L., Benmerah A. (2019). Ciliary kinesins beyond IFT: Cilium length, disassembly, cargo transport and signalling. Biol. Cell.

[4] Dong M., Shi X., Zhou Y., Duan J., He L., Song X., Huang Z., Chen R., Li J., Jia N. (2025). Genetic spectrum and genotype–phenotype correlations in DNAH5-mutated primary ciliary dyskinesia: A systematic review. Orphanet J. Rare Dis..

[5] Olbrich H., Schmidts M., Werner C., Onoufriadis A., Loges N.T., Raidt J., Banki N.F., Shoemark A., Burgoyne T., Al Turki S. (2012). Recessive HYDIN mutations cause primary ciliary dyskinesia without randomization of left-right body asymmetry. Am. J. Hum. Genet..

[6] Loges N.T., Antony D., Maver A., Deardorff M.A., Gulec E.Y., Gezdirici A., Nothe-Menchen T., Hoben I.M., Jelten L., Frank D. (2018). Recessive DNAH9 Loss-of-Function Mutations Cause Laterality Defects and Subtle Respiratory Ciliary-Beating Defects. Am. J. Hum. Genet..

[7] Zhao L., Hou Y., Picariello T., Craige B., Witman G.B. (2019). Proteome of the central apparatus of a ciliary axoneme. J. Cell Biol..

[8] Merveille A.C., Davis E.E., Becker-Heck A., Legendre M., Amirav I., Bataille G., Belmont J., Beydon N., Billen F., Clement A. (2011). CCDC39 is required for assembly of inner dynein arms and the dynein regulatory complex and for normal ciliary motility in humans and dogs. Nat. Genet..

[9] Becker-Heck A., Zohn I.E., Okabe N., Pollock A., Lenhart K.B., Sullivan-Brown J., McSheene J., Loges N.T., Olbrich H., Haeffner K. (2011). The coiled-coil domain containing protein CCDC40 is essential for motile cilia function and left-right axis formation. Nat. Genet..

[10] Loges N.T., Olbrich H., Becker-Heck A., Häffner K., Heer A., Reinhard C., Schmidts M., Kispert A., Zariwal M.A., Leigh M.W. (2009). Deletions and Point Mutations of Cause Primary Ciliary Dyskinesia Due to Dynein Arm Defects. Am. J. Hum. Genet..

[11] Mitchison H.M., Schmidts M., Loges N.T., Freshour J., Dritsoula A., Hirst R.A., O’Callaghan C., Blau H., Al Dabbagh M., Olbrich H. (2012). Mutations in axonemal dynein assembly factor DNAAF3 cause primary ciliary dyskinesia. Nat. Genet..

[12] Tarkar A., Loges N.T., Slagle C.E., Francis R., Dougherty G.W., Tamayo J.V., Shook B., Cantino M., Schwartz D., Jahnke C. (2013). DYX1C1 is required for axonemal dynein assembly and ciliary motility. Nat. Genet..

[13] Horani A., Druley T.E., Zariwala M.A., Pate A.C., Levinson B.T., Van Arendonk L.G., Thornton K.C., Giacalone J.C., Albee A.J., Wilson K.S. (2012). Whole-Exome Capture and Sequencing Identifies Mutation as a Cause of Primary Ciliary Dyskinesia. Am. J. Hum. Genet..

[14] Paff T., Loges N.T., Aprea I., Wu K., Bakey Z., Haarman E.G., Daniels J.M.A., Sistermans E.A., Bogunovic N., Dougherty G.W. (2017). Mutations in PIH1D3 Cause X-Linked Primary Ciliary Dyskinesia with Outer and Inner Dynein Arm Defects. Am. J. Hum. Genet..

[15] Zariwala M.A., Gee H.Y., Kurkowiak M., Al-Mutairi D.A., Leigh M.W., Hurd T.W., Hjeij R., Dell S.D., Chaki M., Dougherty G.W. (2013). Is Mutated in Primary Ciliary Dyskinesia and Interacts with LRRC6. Am. J. Hum. Genet..

[16] Kott E., Duquesnoy P., Copin B., Legendre M., Dastot-Le Moal F., Montantin G., Jeanson L., Tamalet A., Papon J.F., Siffroi J.P. (2012). Loss-of-function mutations in LRRC6, a gene essential for proper axonemal assembly of inner and outer dynein arms, cause primary ciliary dyskinesia. Am. J. Hum. Genet..

[17] Hjeij R., Aprea I., Poeta M., Nothe-Menchen T., Bracht D., Raidt J., Honecker B.I., Dougherty G.W., Olbrich H., Schwartz O. (2023). Pathogenic variants in CLXN encoding the outer dynein arm docking-associated calcium-binding protein calaxin cause primary ciliary dyskinesia. Genet. Med..

[18] Hjeij R., Lindstrand A., Francis R., Zariwala M.A., Liu X., Li Y., Damerla R., Dougherty G.W., Abouhamed M., Olbrich H. (2013). ARMC4 mutations cause primary ciliary dyskinesia with randomization of left/right body asymmetry. Am. J. Hum. Genet..

[19] Onoufriadis A., Paff T., Antony D., Shoemark A., Micha D., Kuyt B., Schmidts M., Petridi S., Dankert-Roelse J.E., Haarman E.G. (2013). Splice-site mutations in the axonemal outer dynein arm docking complex gene CCDC114 cause primary ciliary dyskinesia. Am. J. Hum. Genet..

[20] Hjeij R., Onoufriadis A., Watson C.M., Slagle C.E., Klena N.T., Dougherty G.W., Kurkowiak M., Loges N.T., Diggle C.P., Morante N.F. (2014). CCDC151 mutations cause primary ciliary dyskinesia by disruption of the outer dynein arm docking complex formation. Am. J. Hum. Genet..

[21] Wallmeier J., Shiratori H., Dougherty G.W., Edelbusch C., Hjeij R., Loges N.T., Menchen T., Olbrich H., Pennekamp P., Raidt J. (2016). TTC25 Deficiency Results in Defects of the Outer Dynein Arm Docking Machinery and Primary Ciliary Dyskinesia with Left-Right Body Asymmetry Randomization. Am. J. Hum. Genet..

[22] Schultz R., Elenius V., Fassad M.R., Freke G., Rogers A., Shoemark A., Koistinen T., Mohamed M.A., Lim J.S.Y., Mitchison H.M. (2022). CFAP300 mutation causing primary ciliary dyskinesia in Finland. Front. Genet..

[23] Wu R., Li H., Wu P., Yang Q., Wan X., Wu Y. (2025). LRRC56 deletion causes primary ciliary dyskinesia in mice characterized by dynein arms defects. Biol. Open.

[24] Lucas J.S., Barbato A., Collins S.A., Goutaki M., Behan L., Caudri D., Dell S., Eber E., Escudier E., Hirst R.A. (2017). European Respiratory Society guidelines for the diagnosis of primary ciliary dyskinesia. Eur. Respir. J..

[25] Shoemark A., Frost E., Dixon M., Ollosson S., Kilpin K., Patel M., Scully J., Rogers A.V., Mitchison H.M., Bush A. (2017). Accuracy of Immunofluorescence in the Diagnosis of Primary Ciliary Dyskinesia. Am. J. Respir. Crit. Care Med..

[26] Shoemark A. (2017). Applications of emerging transmission electron microscopy technology in PCD research and diagnosis. Ultrastruct. Pathol..

[27] Raidt J., Wallmeier J., Hjeij R., Onnebrink J.G., Pennekamp P., Loges N.T., Olbrich H., Haffner K., Dougherty G.W., Omran H. (2014). Ciliary beat pattern and frequency in genetic variants of primary ciliary dyskinesia. Eur. Respir. J..

[28] Farley H., Rubbo B., Bukowy-Bieryllo Z., Fassad M., Goutaki M., Harman K., Hogg C., Kuehni C.E., Lopes S., Nielsen K.G. (2018). Proceedings of the 3rd BEAT-PCD Conference and 4th PCD Training School. BMC Proc..

[29] Wallmeier J., Nielsen K.G., Kuehni C.E., Lucas J.S., Leigh M.W., Zariwala M.A., Omran H. (2020). Motile ciliopathies. Nat. Rev. Dis. Primers.

[30] Chen W., Guo Z., Li M., Sheng W., Huang G. (2024). Next-Generation Sequencing-Based Copy Number Variation Analysis in Chinese Patients with Primary Ciliary Dyskinesia Revealed Novel DNAH5 Copy Number Variations. Phenomics.

[31] Emiralioglu N., Taskiran E.Z., Kosukcu C., Bilgic E., Atilla P., Kaya B., Gunaydin O., Yuzbasioglu A., Tugcu G.D., Ademhan D. (2020). Genotype and phenotype evaluation of patients with primary ciliary dyskinesia: First results from Turkey. Pediatr. Pulmonol..

[32] Dougherty G.W., Mizuno K., Nothe-Menchen T., Ikawa Y., Boldt K., Ta-Shma A., Aprea I., Minegishi K., Pang Y.P., Pennekamp P. (2020). CFAP45 deficiency causes situs abnormalities and asthenospermia by disrupting an axonemal adenine nucleotide homeostasis module. Nat. Commun..

[33] Al-Mutairi D.A., Alsabah B.H., Alkhaledi B.A., Pennekamp P., Omran H. (2022). Identification of a novel founder variant in DNAI2 cause primary ciliary dyskinesia in five consanguineous families derived from a single tribe descendant of Arabian Peninsula. Front. Genet..

[34] Guo T., Lu C., Yang D., Lei C., Liu Y., Xu Y., Yang B., Wang R., Luo H. (2022). Case Report: DNAAF4 Variants Cause Primary Ciliary Dyskinesia and Infertility in Two Han Chinese Families. Front. Genet..

[35] Goutaki M., Meier A.B., Halbeisen F.S., Lucas J.S., Dell S.D., Maurer E., Casaulta C., Jurca M., Spycher B.D., Kuehni C.E. (2016). Clinical manifestations in primary ciliary dyskinesia: Systematic review and meta-analysis. Eur. Respir. J..

[36] Hannah W.B., Seifert B.A., Truty R., Zariwala M.A., Ameel K., Zhao Y., Nykamp K., Gaston B. (2022). The global prevalence and ethnic heterogeneity of primary ciliary dyskinesia gene variants: A genetic database analysis. Lancet Respir. Med..

[37] Zhao X., Bian C., Liu K., Xu W., Liu Y., Tian X., Bai J., Xu K.-F., Zhang X. (2021). Clinical characteristics and genetic spectrum of 26 individuals of Chinese origin with primary ciliary dyskinesia. Orphanet J. Rare Dis..

[38] Poplawska K., Griffiths A., Temme R., Adamko D.J., Nykamp K., Shapiro A.J. (2023). Deletions in DNAL1 Cause Primary Ciliary Dyskinesia Across North American Indigenous Populations. J. Pediatr..

[39] Rodriguez N.M., Schreck L.D., Pedersen E.S.L., Cizeau I., Müller L., Kruljac C., Lucas J.S., Goutaki M., Kuehni C.E. (2023). Diagnostic testing in people with primary ciliary dyskinesia: An international participatory study. PLoS Glob. Public Health.

[40] Pedersen E.S.L., Goutaki M., Schreck L.D., Rindlisbacher B., Dixon L., Lucas J.S., Kuehni C.E. (2024). Questionnaire-assessed genotypes and associations with symptoms in primary ciliary dyskinesia. ERJ Open Res..

[41] Baz-Redon N., Rovira-Amigo S., Fernandez-Cancio M., Castillo-Corullon S., Cols M., Caballero-Rabasco M.A., Asensio O., Martin de Vicente C., Martinez-Colls M.D.M., Torrent-Vernetta A. (2020). Immunofluorescence Analysis as a Diagnostic Tool in a Spanish Cohort of Patients with Suspected Primary Ciliary Dyskinesia. J. Clin. Med..

[42] Ta-Shma A., Hjeij R., Perles Z., Dougherty G.W., Abu Zahira I., Letteboer S.J.F., Antony D., Darwish A., Mans D.A., Spittler S. (2018). Homozygous loss-of-function mutations in MNS1 cause laterality defects and likely male infertility. PLoS Genet..

[43] Yue Y., Huang Q., Zhu P., Zhao P., Tan X., Liu S., Li S., Han X., Cheng L., Li B. (2019). Identification of Pathogenic Mutations and Investigation of the NOTCH Pathway Activation in Kartagener Syndrome. Front. Genet..

[44] Olbrich H., Cremers C., Loges N.T., Werner C., Nielsen K.G., Marthin J.K., Philipsen M., Wallmeier J., Pennekamp P., Menchen T. (2015). Loss-of-Function GAS8 Mutations Cause Primary Ciliary Dyskinesia and Disrupt the Nexin-Dynein Regulatory Complex. Am. J. Hum. Genet..

[45] Jeanson L., Thomas L., Copin B., Coste A., Sermet-Gaudelus I., Dastot-Le Moal F., Duquesnoy P., Montantin G., Collot N., Tissier S. (2016). Mutations in GAS8, a Gene Encoding a Nexin-Dynein Regulatory Complex Subunit, Cause Primary Ciliary Dyskinesia with Axonemal Disorganization. Hum. Mutat..

[46] Zlotina A., Barashkova S., Zhuk S., Skitchenko R., Usoltsev D., Sokolnikova P., Artomov M., Alekseenko S., Simanova T., Goloborodko M. (2024). Characterization of pathogenic genetic variants in Russian patients with primary ciliary dyskinesia using gene panel sequencing and transcript analysis. Orphanet J. Rare Dis..

[47] Nussbaumer M., Kieninger E., Tschanz S.A., Savas S.T., Casaulta C., Goutaki M., Blanchon S., Jung A., Regamey N., Kuehni C.E. (2021). Diagnosis of primary ciliary dyskinesia: Discrepancy according to different algorithms. ERJ Open Res..

[48] Guo S., Tang D., Chen Y., Yu H., Gu M., Geng H., Fang J., Wu B., Ruan L., Li K. (2024). Association of novel DNAH11 variants with asthenoteratozoospermia lead to male infertility. Hum. Genom..

[49] Maddirevula S., Awartani K., Coskun S., AlNaim L.F., Ibrahim N., Abdulwahab F., Hashem M., Alhassan S., Alkuraya F.S. (2020). A genomics approach to females with infertility and recurrent pregnancy loss. Hum. Genet..

[50] Schreck L.D., Pedersen E.S.L., Dexter K., Manion M., Bellu S., Cizeau I., Dexter K., Dixon L., Fernández T.L., Grieder S. (2024). Infertility and pregnancy outcomes among adults with primary ciliary dyskinesia. Hum. Reprod. Open.

[51] Frommer A., Hjeij R., Loges N.T., Edelbusch C., Jahnke C., Raidt J., Werner C., Wallmeier J., Grosse-Onnebrink J., Olbrich H. (2015). Immunofluorescence Analysis and Diagnosis of Primary Ciliary Dyskinesia with Radial Spoke Defects. Am. J. Respir. Cell Mol. Biol..

[52] Mabrouk I., Al-Harthi N., Mani R., Montantin G., Tissier S., Lagha R., Ben Abdallah F., Hassan M.M., Alhomrani M., Gaber A. (2022). Combining RSPH9 founder mutation screening and next-generation sequencing analysis is efficient for primary ciliary dyskinesia diagnosis in Saudi patients. J. Hum. Genet..

[53] Shoemark A., Boon M., Brochhausen C., Bukowy-Bieryllo Z., De Santi M.M., Goggin P., Griffin P., Hegele R.G., Hirst R.A., Leigh M.W. (2020). International consensus guideline for reporting transmission electron microscopy results in the diagnosis of primary ciliary dyskinesia (BEAT PCD TEM Criteria). Eur. Respir. J..

[54] Fassad M.R., Patel M.P., Shoemark A., Cullup T., Hayward J., Dixon M., Rogers A.V., Ollosson S., Jackson C., Goggin P. (2020). Clinical utility of NGS diagnosis and disease stratification in a multiethnic primary ciliary dyskinesia cohort. J. Med. Genet..

[55] Horani A., Ferkol T.W., Dutcher S.K., Brody S.L. (2016). Genetics and biology of primary ciliary dyskinesia. Paediatr. Respir. Rev..

