# Supplementary figures and images for: Diagnostic Role of Immunofluorescence Analysis in Primary Ciliary Dyskinesia-Suspected Individuals
Source: J Clin Med. 2025 Mar 13;14(6):1941. doi: 10.3390/jcm14061941 (PMC11942966; doi:10.3390/jcm14061941)

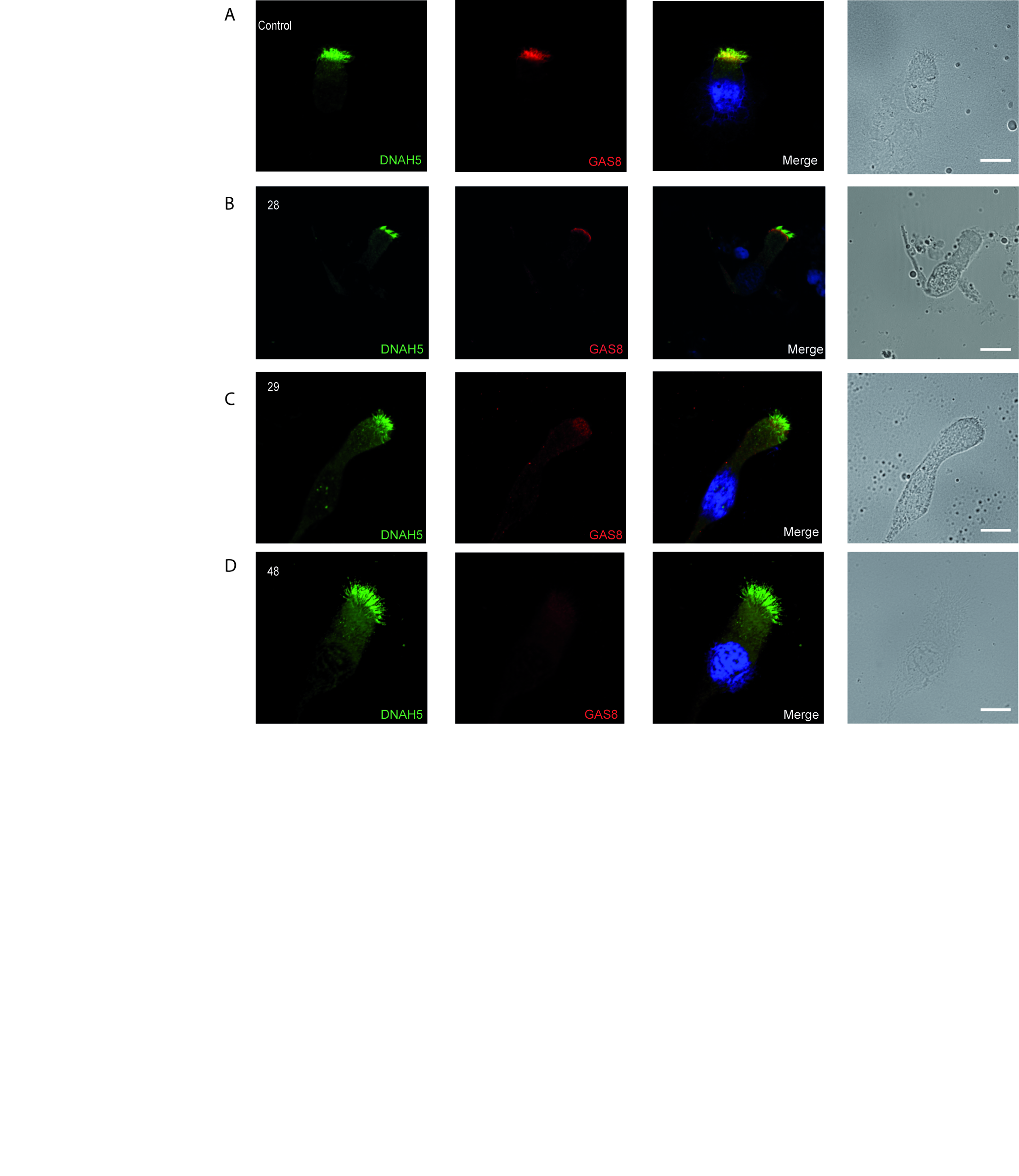

Supplement: Supplementary file 1 [file jcm-14-01941-s001.zip › revised supplementary material/Figure S2.tif]

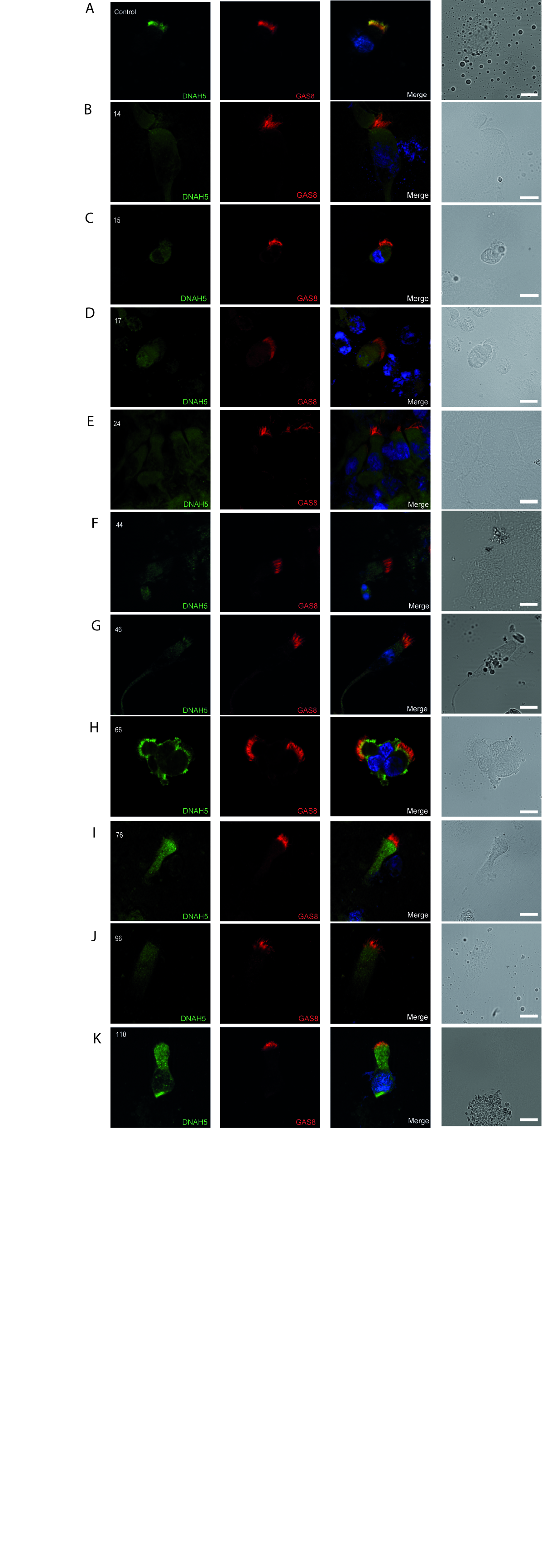

Supplement: Supplementary file 1 [file jcm-14-01941-s001.zip › revised supplementary material/Figure S1.tif]
